# Graphene Oxide and Methylglyoxal: a combined strategy against chronic wound pathogens

**DOI:** 10.1007/s00253-026-14050-2

**Published:** 2026-09-24

**Authors:** Silvia Di Lodovico, Antonella Fontana, Paola Di Fermo, Firas Diban, Emanuela Di Campli, Serena Pilato, Simonetta D’Ercole, Luigina Cellini, Mara Di Giulio

**Affiliations:** 1 Department of Pharmacy, University “G. d’Annunzio” Chieti-Pescara, Via Dei Vestini, 31, Chieti, 66100 Italy; 2 Department of Medical, Oral and Biotechnological Sciences, University “G. d’Annunzio” Chieti-Pescara, Via Dei Vestini, 31, Chieti, 66100 Italy

**Keywords:** Graphene Oxide and Methylglyoxal combination, *Staphylococcus aureus*, *Pseudomonas aeruginosa*, Chronic wound infections, Informing and mature LCWBs

## Abstract

Chronic wound infections are difficult to treat due to the polymicrobial biofilms that delay wound healing increasing antimicrobial tolerance and limiting patient compliance. To address this challenge, we developed a non-antibiotic combination of Graphene Oxide (GO) and Methylglyoxal (MGO) (patent N. 102022000024408-Composizione per il trattamento delle infezioni a carico delle lesioni cutanee) against clinically relevant chronic wound pathogens. For the tests, clinical antimicrobial resistant *Staphylococcus aureus* and *Pseudomonas aeruginosa* were used. MGO MIC was determined and the best GO + MGO combination, evaluated by using checkboard test, was tested in terms of: i) fluid membrane changes; ii) *S. aureus* and *P. aeruginosa* CFUs/mg reduction in Lubbock Chronic Wound Biofilm-LCWB, a recognized polymicrobial in vitro chronic wound biofilm model; iii) *P. aeruginosa* motility. MGO MIC values ranged from 32 to 128 mg/l. Notably, synergistic interactions between GO and MGO were observed exclusively against *S. aureus*, whereas the combinations showed additive effects against *P. aeruginosa*. The best GO + MGO (6.25 + 64 mg/l) combination increased bacterial membrane fluidity, inhibited informing and mature LCWBs by 60–80% in terms of CFU/mg and *P. aeruginosa* twitching motility. GO + MGO combination exhibited an additive antimicrobial/antibiofilm activity with a multi-target action. Overall, the GO + MGO combination, at recognized non-toxic concentrations, is a valid and innovative non-antibiotic solution for wound management affecting the polymicrobial chronic wound biofilms and *P. aeruginosa* motility.

**Supplementary Information:**

The online version contains supplementary material available at https://doi.org/10.1007/s00253-026-14050-2.

## Introduction

Chronic wounds are wounds that fail to heal completely within the normal timeframe, from four weeks to three months, and they are characterized by persistent infections (Eriksson et al. 2022; Graves et al. 2021). Nowadays, they represent a serious and “silent epidemic” issue that significantly affects patient’s quality of life due to prolonged healing time, recurrent infections, chronic pain, depression and reduced mobility (Graves et al. 2021). Wound healing is a multifactorial complex and dynamic biological process involving multiple cellular and molecular events that must be tightly coordinated to ensure efficient tissue repair (Tottoli et al. 2020; Borges et al. 2024). It depends on both host-related (like age, genetics, chronic diseases such as diabetes, malnutrition, and poor systemic conditions) and microbial factors (pathogens causing infection and microbiota dysbiosis) that significantly influence the healing outcomes (Sen 2021; Diban et al. 2023).

The microbial colonization plays a critical role in wound chronicity, affecting wound’s healing and treatment efficacy (Diban et al. 2023). In fact, wound healing is frequently hindered by bacterial infections associated with polymicrobial biofilms. Within these structured microbial communities, bacteria are encased in self-produced Extracellular Polymeric Substance (EPS) matrix and exhibit lower metabolic activity compared with their planktonic counterparts. These characteristics contribute to increased microbial tolerance toward antimicrobial treatments, delaying tissue regeneration and the persistence of refractory ulcers (Deglovic et al. 2022). Moreover, biofilm-associated bacteria display distinct metabolic adaptations that enhance their survival in the highly stressful chronic-wound microenvironment, which is defined by immune pressure, fluctuating oxygen levels, and limited nutrient availability (Almuhanna 2025).

Among the microorganisms isolated from chronic wounds, *Staphylococcus aureus* and *Pseudomonas aeruginosa* are the predominant species and are strongly associated with impaired healing and biofilm formation (Ersanli et al. 2023; Pouget et al. 2022).

The interaction between *Pseudomonas aeruginosa* and *Staphylococcus aureus* is initially characterized by an antagonistic relationship, in which *P. aeruginosa* predominates over *S. aureus.* As the infection progresses, this interaction shifts toward a synergistic phase leading to a coaggregation within a polymicrobial biofilm (Vestweber et al. 2024). This coexistence increases the antimicrobial tolerance and contributes to delayed wound healing (Uberoi et al. 2024). Alginate production by *P. aeruginosa* has been shown to promote coexistence with *S. aureus* while the immune protein calprotectin induces the co-colonization of both pathogens. Furthermore, Vestweber et al (2024) demonstrated that *S. aureus* acts as a pioneer facilitating and aiding the adhesion of *P. aeruginosa* that, in turn, promotes an invasive phenotype of *S. aureus.*

In chronic wounds, colonization by *S. aureus* and *P. aeruginosa* is not uniformly mixed but instead exhibits a specific spatial microbial distribution. Specifically, *S. aureus* is located in the superficial regions of the wound whereas *P. aeruginosa* is typically found in deeper tissue layers (Ibberson et al. 2022).

The Lubbock Chronic Wound Biofilm (LCWB) model is a well-established in vitro system that recapitulates key features of chronic wound multispecies biofilms, including their three-dimensional organization and spatial microbial distribution (Kucera et al. 2014; Thaarup and Bjarnsholt 2021). Its blood- and plasma-containing environment supports the coexistence of *S. aureus* and *P. aeruginosa*, with *S. aureus*-mediated coagulation promoting the formation of a fibrin network that provides a scaffold for *P. aeruginosa* adhesion and biofilm development. By reproducing both the polymicrobial nature and the structural complexity of chronic wound biofilms within a wound-like microenvironment, the LCWB model provides a physiologically relevant platform for assessing antimicrobial efficacy and discriminating the most promising therapeutic approaches under conditions that more closely reflect the clinical setting than conventional planktonic or monospecies biofilm assays.

Several approaches are currently employed to control microbial proliferation and biofilm formation in chronic wounds. Among these, silver-based therapies, used either alone or in combination with antibiotics, represent a cornerstone of wound management (Norman et al. 2017). Antibiotic treatment remains essential in cases of clinically infected wounds, presenting signs such as purulent drainage, erythema, warmth, pain, tenderness, or induration (Di Bella et al. 2024). However, growing concerns regarding antimicrobial resistance and treatment-associated adverse effects have promoted the development of non-antibiotic therapeutic approaches aimed at preserving antimicrobial efficacy while minimizing the limitations associated with conventional antibiotic use (Brown 2015).

To address the growing challenge of chronic wound infections through non-antibiotic approaches, this study focuses on the evaluation of Graphene Oxide (GO) and Methylglyoxal (MGO) as an alternative antimicrobial combination.

Graphene Oxide (GO) has attracted considerable attention as an antimicrobial nanomaterial; indeed, GO presented significant capability to inhibit the growth of *Escherichia coli, S. aureus*, *P. aeruginosa* and *Candida albicans*. This action is attributed to several possible mechanisms including the direct wrapping action of bacterial cells, its ability to disrupt bacterial membranes, Reactive Oxygen Species (ROS) production and pore formation (Gurunathan et al. 2012; Ravikumar et al. 2022; Liu et al. 2011). Importantly, GO has been shown to enhance the antibacterial efficacy of several antimicrobial agents through increased bacterial membrane permeability, improved local delivery, and synergistic physicochemical interactions. Indeed, Edwin et al. (2025) highlighted the multifunctional potential of GO-based materials for wound healing, showing that, when combined with various metallic nanoparticles, GO may contribute not only to antibacterial activity but also to anti-inflammatory, antioxidant, and regenerative effects (Edwin et al. 2025).

In a previous study, we demonstrated the antimicrobial and antibiofilm action of GO against microorganisms isolated from skin chronic wounds (Di Giulio et al. 2020). GO is an amphiphilic two-dimensional material characterized by a large specific surface area and a heterogeneous surface chemistry, in which oxygen-containing functional groups confer hydrophilicity and enable hydrogen bonding and electrostatic interactions with the aqueous environment and biomolecules, while residual aromatic sp^2^ domains can establish hydrophobic and π–π interactions with proteins, polymers and cellular membranes. Due to these features it is able to negatively interfere with microbial growth, their aggregation and adhesion (Di Giulio et al. 2020). In addition, GO showed a significant effect against *S. aureus* and *P. aeruginosa* growth in the LCWB model (Di Giulio et al. 2020). In presence of GO, the fibrin network appears looser and probably this effect facilitates the permeability of other compounds in the LCWB by increasing the antimicrobial agent effectiveness (Park et al. 2008; Di Giulio et al. 2020).

On the other hand, MGO is a reactive dicarbonyl compound that is produced endogenously in the body and has been widely identified in both animal and plant tissues (McArthur et al. 1997). In addition, MGO is a naturally occurring bioactive compound in Manuka honey, a monofloral honey, produced by *Apis mellifera* from the nectar of Manuka tree flowers in New Zealand. It has been used since ancient times in traditional medicine, particularly for wound healing and the restoration of damaged epithelial barriers (Majtan 2020; Bucekova et al. 2019). The Manuka honey antimicrobial action is attributed to the presence of Methylglyoxal (MGO) that is presented in a concentration of 38–829 mg/kg. It was found that the MGO levels of Manuka honey are 20-fold higher in comparison to other non-Manuka honeys.

Recent studies demonstrate that Manuka honey inhibits the growth and biofilm formation of *S. aureus* (including Methicillin-resistant *S. aureus* - MRSA), *Streptococcus pyogenes*, *P. aeruginosa*, and *Escherichia coli*, even in multidrug-resistant strains (Roberts et al. 2021; Lu et al. 2014; Paramasivan et al. 2014; Combarros-Fuertes et al. 2019). MGO can enhance the efficacy of traditional drugs against *S. aureus.* Hayes et al. (2018) demonstrated that MGO increased intracellular accumulation of linezolid, an antibiotic belonging to the oxazolidinone class, used to treat bacterial infections, particularly those caused by *S. aureus* resistant to more conventional antibiotics, in MRSA strains increasing its antibiotic effect. In addition, MGO has shown a significant antibiofilm action against skin wound infections (Lu et al. 2014).

Although GO and MGO have individually demonstrated antimicrobial and antibiofilm properties, their combined activity against clinically relevant polymicrobial chronic wound biofilms remains unexplored. Their different mechanisms of action suggest these compounds may exert complementary biological effects. GO may act primarily through direct physical interactions with microbial cells and has been shown to interfere with fibrin biofilm architecture, potentially facilitating the penetration of antimicrobial compounds into the biofilm. Furthermore, its high surface area and abundant oxygen-containing functional groups of GO may promote MGO adsorption and localized delivery at the bacterial surface. Unlike GO, MGO exerts its antimicrobial activity through its high electrophilic reactivity, interacting with essential cellular macromolecules, including proteins and nucleic acids, thereby impairing bacterial cellular functions (Lee and Park 2017). Therefore, combining these two compounds could represent a promising non-antibiotic strategy for simultaneously disrupting biofilm architecture and targeting bacterial cells through complementary mechanisms of action. Based on these considerations, the aim of this work was to evaluate the antimicrobial and anti-virulence activities of GO combined with MGO against *S. aureus* PECHA 10 and *P. aeruginosa* PECHA 4 in dual-species LCWB model. Specifically, the study was designed to: (i) investigate the effects of GO and MGO, alone and in combination, on bacterial membrane fluidity, (ii) assess their efficacy against dual-species LCWB biofilms using quantitative and microscopic analyses, and (iii) investigate their anti-virulence effect on *P. aeruginosa* swimming, swarming and twitching motility.

## Materials and methods

### Bacterial cultures

*Staphylococcus aureus* PECHA 10 and *P. aeruginosa* PECHA 4, isolated from chronic wounds (Di Giulio et al. 2020) (ID n. richycnvw), were used in this study. For the experiments, bacteria were cultured in Trypticase Soy Broth (TSB, Oxoid, Milan, Italy) and the broth cultures were adjusted to an optical density at 600 nm (OD_600_) of 0.125 (Di Giulio et al. 2020).

### Graphene Oxide (GO) and Methylglyoxal solution (MGO)

The preparation of Graphene Oxide (GO, Graphenea, Donostia San Sebastian, Spain) aqueous dispersion was performed according to Di Giulio et al. (2020) and Di Giulio et al. (2018). Briefly, a 4 g/l aqueous solution of GO was diluted with PBS to achieve the target concentration. The mixture was ultrasonicated for 10 min and sterilized under UVC light (at λ 250 nm) for 2 h. GO concentration was measured spectrophotometrically at λ 230 nm, and flake size was analyzed using dynamic light scattering (DLS). Methylglyoxal (MGO) solution (40% in water) was purchased from Sigma Aldrich (Milan, Italy) and diluted in PBS to obtain the requested concentrations. Importantly, previous studies have reported no significant cytotoxic effects of GO (Bengtson et al. 2016; Ettorre et al. 2016) and MGO (D’Amico et al. 2025; Di Fermo et al. 2025) on different human cell models at the concentrations and under the experimental conditions investigated, supporting their potential compatibility with wound-related applications.

### FT-IR and Raman spectroscopy characterization of GO dispersion

Fourier-transform infrared (FTIR) and Raman spectroscopy analyses were performed on the GO dispersion before and after UVC treatment to evaluate potential modifications induced by the sterilization process. The samples were obtained by dropcasting the 1 mg/ml GO dispersion on a silicon wafer or on diamond crystal and letting the samples evaporate. IR spectra were obtained using a Shimadzu IRAffinity-1S FTIR spectrophotometer (Shimadzu Italia S.r.l., Milan, Italy) equipped with a sealed and desiccated interferometer, a DLATGS (Deuterated Triglycine Sulphate Doped with L-Alanine) detector and a single reflection diamond ATR crystal (QATR 10, Shimadzu Italia S.r.l., Milan, Italy). Spectra were recorded over 400–4000 cm^−1^ by co-adding 45 interferograms at an 8 cm^−1^ resolution with Happ-Genzel apodization. The ATR crystal was carefully cleaned before each analysis, a background spectrum was recorded for each sample, and all measurements were performed in triplicate. Spectral processing was carried out using LabSolution IR software, version 2.27 (Shimadzu Italia S.r.l., Milan, Italy). The Raman spectra of GO dispersions, before and after UVC treatment, were obtained by confocal and high-performance Raman microscopy (XploRA PLUS, HORIBA, Japan) with deep-cooled CCD detector technology. LabSpec (Horiba, Japan) was employed to control the Raman spectroscopic system and for the optimization and processing of the acquired data. All Raman spectroscopic measurements were performed in the range 250–3400 cm^−1^ and with an 1800-line/mm grating. The samples were analyzed by using a 532 nm laser, with 10 s acquisition time and 10 accumulations. Moderate power irradiation at the sample surface was used (~ 10 mW), focusing onto the sample surface with a 50× objective to avoid laser-induced heating.

### Methylglyoxal solution (MGO) susceptibility assay

The MGO MIC was performed against *S. aureus* PECHA 10 and *P. aeruginosa* PECHA 4 by the microdilution method according to CLSI guidelines (CLSI 2019). Twofold dilutions of MGO stock solution ranging from 512 mg/l to 4 mg/l were performed in cation-adjusted Mueller–Hinton Broth II (MHB2, Oxoid, Milan, Italy). For the MIC determination, 100 µl of MGO and 100 µl of each broth culture (10^5^ CFU/ml) were dispensed in each well of 96-well microtiter plate and incubated in aerobic condition at 37 °C. MICs were determined as the lowest concentration of MGO able to inhibit the visible growth of the microorganisms. MBCs were determined by sub-culturing 10 μl of suspensions from the non-turbid wells on Mueller–Hinton (MHA, Oxoid, Milan, Italy) agar and incubated as described above. The MBC represents the lowest concentration of MGO that inhibits bacterial growth on agar plates. As negative control, MGO dilutions in MHB2 without bacteria were tested.

### Checkerboard assay

To demonstrate the antimicrobial action of the GO + MGO combination, the synergisms between GO and MGO were determined by the checkerboard test (Diban et al. 2025). The GO concentrations used in this test ranged from 50 mg/l to 0.78 mg/l and the MGO concentration ranged from 250 mg/l to 1 mg/l. The dilutions of the two substances were inoculated in microtiter plates and incubated as described above. The checkerboard test was used to calculate the Fractional Inhibitory Concentration (FIC), that is equal to MIC_AB_/MIC_A_ + MIC_BA_/MIC_B_, where MIC_AB_ is the MIC of compound A in presence of compound B; MIC_BA_ is the MIC of B in presence of A. FIC Index (FIC I) values were interpreted according to Odds (2003) namely synergism FIC I ≤ 0.5, antagonism FIC I ≥ 4.0, and additive FIC I > 0.5–4.0. As negative control, GO and MGO dilutions without bacteria were tested.

### Membrane fluidity assessment

To evaluate membrane fluidity in *S. aureus* PECHA 10 and *P. aeruginosa* PECHA 4 in the presence of the GO + MGO combination, Laurdan generalized polarization (GP_exc_) was measured (Di Lodovico et al. 2020). The effect was determined with GO at 50 mg/l and MGO at concentrations of 2 × MIC, MIC, 1/2 MIC both individually and in combination with GO. For the analysis Laurdan at 10 µM was used and emission spectra were obtained in a Varian Cary Eclipse fluorescence spectrofluorometer (Agilent Technologies, Santa Clara, California, USA) at an excitation wavelength of 350 nm and emission wavelengths collected from 410 to 550 nm. The excitation GP was calculated using the following equation: GPexc = (I_440_ − I_490_)/(I_440_ + I_490_), where I_440_ and I_490_ are fluorescence intensities at 440 and 490 nm, respectively. Each determination was performed in duplicate for three independent experiments. As positive control, Ciprofloxacin was used (data not shown). GP_exc_ values were analyzed using one-way ANOVA followed by Tukey’s post hoc test.

### GO + MGO combination on LCWB model

The effect of the best GO + MGO combination (6.25 mg/l GO + 64 mg/l MGO, characterized FIC I = 0.63 for *P. aeruginosa* PECHA 4) was evaluated in informing and mature LCWB model (Di Giulio et al. 2020). The chosen combination derives from the checkerboard test against *P. aeruginosa* which was the most resistant strain against GO and MGO. In addition, the combination was also suitable for *S. aureus* with which synergies were detected. Briefly, for informing LCWB, 100 μl of GO at final concentration of 50 mg/l, or 100 μl of MGO at final concentration of 1/2 MIC value of the strain more susceptible (*S. aureus *PECHA 10) to MGO (16 mg/l) or 100 μl of final concentration of 1/2 of the additive combination of GO + MGO or 100 μl of phosphate-buffered saline (PBS) (for the control) were added to the LCWB medium. The used glass test tubes were incubated for 48 h at 37 °C. As positive control, Amikacin was used (data not shown).

For the mature LCWB, after 48 h of incubation, the mature biofilm was placed on Petri dishes containing Bolton Broth (Oxoid, Milan, Italy) with 1.5% bacteriological agar to produce the “wound bed” for the chronic wound biofilm model (Di Giulio et al. 2020) and treated with GO at a final concentration of 50 mg/l or with MGO at final concentration of 2 MIC value of the strain more resistant (*P. aeruginosa* PECHA 4) to MGO (256 mg/l), or with 2 or 4 times of the additive combination of GO + MGO or PBS (for the control) and incubated for 24 h at 37 °C.

After incubation, the informing and mature biofilms were harvested, washed two times with sterile PBS and the weight was measured by using an analytical balance. Subsequently, the biofilms were vortexed for 2 min, sonicated for 3 min (with an ultrasound bath), vortexed again for 2 min and diluted in PBS for the microbial enumeration on Mannitol Salt Agar (Oxoid, Milan, Italy, MSA) and on Cetrimide Agar (Oxoid, Milan, Italy, CET). The antimicrobial effect was determined as percentage reduction of *S. aureus* PECHA 10 and *P. aeruginosa* PECHA 4 CFUs per mg of LCWB with respect to the control.

Each determination was performed at least in triplicate for three independent experiments. Percentages of CFU/mg reduction were analyzed using one-way ANOVA followed by Tukey’s post hoc test.

The effect of bacterial viability of GO, MGO, and GO + MGO combination was also evaluated by fluorescence microscopy. After treatment, the harvested and washed informing and mature LCWBs, as described above, were centrifuged at 10.000 rpm for 5 min and the pellet was resuspended with Live/Dead staining solution (Molecular Probes Inc., Invitrogen, San Giuliano Milanese, Italy) and visualized under a Leica 4000 DM microscope.

The GO + MGO combination effect was also evaluated by Scanning Electron Microscopy (SEM) according to Di Giulio et al (2020). Briefly, the samples were fixed in a glutaraldehyde solution, dehydrated in ethanol solutions and then immersed in hexamethyldisilazane (HMDS, Sigma Aldrich, Milan, Italy). The dried samples were subjected to gold-sputtering with a Desk Sputter Coater (Phenom-WorldB.V., Eindhoven, 5652, AM, Netherlands) and then observed by SEM (Phenom-WorldB.V., Dillenburgstra at 97 Eindhoven, 5652, AM, Netherlands).

### Swimming, swarming and twitching motility

The effect of GO and MGO on the reduction of *P. aeruginosa* swimming, swarming and twitching motilities was evaluated. For the swarming assays, *P. aeruginosa* PECHA 4 overnight cultures were inoculated at the center of Petri plates containing 1% peptone, 0.5% NaCl, 0.5% agar and 0.5% filter-sterilized D-glucose with or without GO at a final concentration of 50 mg/l, 1/2 MIC of MGO (64 mg/l), 1/2 of additive combination of GO + MGO (3.12 mg/l + 32 mg/l). The plates were incubated at 37 °C for 24 h (Di Lodovico et al. 2025). The swarming migration was recorded by monitoring the migration of the bacterial swarm fronts. For swimming motility assay, *P. aeruginosa* PECHA 4 overnight cultures were inoculated at the center of the plates containing 1% tryptone, 0.5% NaCl and 0.3% agar and with or without GO at a final concentration of 50 mg/l, 1/2 MIC of MGO (64 mg/l), 1/2 of additive combination of GO + MGO (3.12 mg/l + 32 mg/l)**.** The plates were incubated at 37 °C for 24 h (Di Lodovico et al. 2025). The swimming migration was recorded by monitoring swim zones of the bacterial cells after 24 h (Di Lodovico et al. 2025). For the twitching, the standardized broth cultures were inoculated with or without GO at final concentration of 50 mg/l, 1/2 MIC of MGO (64 mg/l), 1/2 of additive combination of GO + MGO (3.12 mg/l + 32 mg/l) to the bottom of the twitching plates consisting of 10 g/l tryptone, 5 g/l yeast extract, 10 g/l NaCl, 1% agar. GO and MGO alone and in combination, were added to 3.5 ml of medium at the final requested concentration. For each motility test, as a control group, the sample was inoculated in the plates without GO or MGO. The plates were incubated at 37 °C for 24 h and then the agar was removed and the halo was stained with 0.1% Crystal Violet and measured (Di Lodovico et al. 2025). Each test was repeated in triplicate for three independent experiments. Halos values (cm) were analysed using one-way ANOVA followed by Tukey’s post hoc test.

### Statistical analysis

Differences between controls and experimental groups, as well as among the treatment conditions, were evaluated using one-way ANOVA followed by Tukey’s post hoc test. *P* ≤ 0.05 was considered statistically significant.

## Results

### GO dispersion characterization

Prior to its use for antibacterial assays, the GO dispersion was subjected to UVC treatment under aseptic conditions. Since UVC irradiation may potentially induce structural or chemical modification in carbon-based nanomaterials, Raman and FTIR spectroscopy were performed before and after the sterilization treatment to verify the preservation of the physicochemical properties of GO. As reported in Figure S1 of Supplementary Materials, the combined Raman and FTIR analyses demonstrated that the UVC treatment preserved both the structural organization and chemical composition of the GO dispersion, with no statistically detectable alteration in the main vibrational features of GO after treatment.

### MGO susceptibility and checkerboard assays

The MIC value of MGO was 32 mg/l and 128 mg/l for *S. aureus* and *P. aeruginosa*, respectively. The MBC values were equal to MICs for both strains (Table 1).

**Table 1:** MICs and MBCs of MGO against *S. aureus* PECHA 10 and *P. aeruginosa* PECHA 4

Strains	MGO (mg/l)			
	1/2 MIC	MIC	2 MIC	MBC
*S. aureus* PECHA 10	16	32	64	32
*P.* *aeruginosa* PECHA 4	64	128	256	128

In the checkerboard assay, synergisms between GO and MGO (FIC Index ≤ 0.5) were observed for *S. aureus* PECHA 10 resulting in a two-to four-fold reduction in the GO concentration [50 mg/l, a previously demonstrated bacteriostatic concentration (Di Giulio et al. 2018)] and a two-to three-fold reduction in the MGO MIC. An additive effect of GO and MGO (FIC Index > 0.5–4.0) was observed in presence of *P. aeruginosa* PECHA 4 resulting in a two-to three-fold reduction in the GO MIC and an onefold reduction of MGO MIC. The best FIC Index values were 0.18 (3.12 mg/l GO + 4 mg/l MGO) for *S. aureus* PECHA 10 and 0.63 (6.25 mg/l of GO + 64 mg/l MGO) for *P. aeruginosa* PECHA 4 (Table 2).

**Table 2:** Synergistic and additive combinations between GO and MGO and FIC Index values against *S. aureus* PECHA 10 and *P. aeruginosa* PECHA 4 (synergistic combinations = FIC I ≤ 0.5; additive combinations = FIC I > 0.5–4.0; antagonistic combinations = FIC I ≥ 4.0)

Strains	Synergistic combinations (GO + MGO) mg/l	Additive combinations (GO + MGO) mg/l	FIC index
*S. aureus *PECHA 10	12.5 + 4		0.37
	6.25 + 4		0.25
	3.12 + 4		0.18
	12.5 + 8		0.50
	6.25 + 8		0.37
	3.12 + 8		0.31
*P. aeruginosa *PECHA 4		50 + 64	1.51
		25 + 64	1.01
		12.5 + 64	0.76
		6.25 + 64	0.63

For the experiments assessing the GO + MGO effect, we used the best additive combination (6.25 mg/l GO + 64 mg/l MGO) determined in *P. aeruginosa* PECHA 4, as this combination was also appropriate for *S. aureus*.

### Membrane fluidity assessment

The membrane fluidity was determined by using GP_exc_ values. In presence of MGO at concentrations corresponding to 2 × MIC (*p* < 0.05) or MIC or 1/2 MIC, a decrease in GP_exc_ value was detected in *S. aureus* with respect to the control. GP_exc_ values for *P. aeruginosa* remained unchanged across all tested MGO (Table 3). A decrease of GP_exc_ values is indicative of an increase in membrane fluidity. Although GO interference with fluorescence signals limited the reliability of GP_exc_ measurements for assessing membrane fluidity, a clear decrease in GP_exc_ values was observed in *S. aureus* upon combined GO+MGO treatment, suggesting an increase in membrane fluidity.

**Table 3:** GP_exc_ values ± SD of *S. aureus* PECHA 10 and *P. aeruginosa* PECHA 4 treated with GO at 50 mg/l, MGO and GO + MGO (6.25 mg/l + 64 mg/l). ^#^statistically significant (*p* < 0.05) in respect to the control

GP_exc_ values						
Strains	Control	GO	MGO			GO + MGO
			2 × MIC	MIC	1/2 MIC	
*S. aureus* PECHA 10	0.453 ± 0.07	0.283^#^ ± 0.07	0.324^#^ ± 0.00	0.332 ± 0.04	0.375 ± 0.02	0.301^#^ ± 0.06
*P. aeruginosa *PECHA 4	0.188 ± 0.03	0.161 ± 0.06	0.174 ± 0.02	0.166 ± 0.02	0.166 ± 0.05	0.168 ± 0.03

### GO and MGO combination on LCWB model

**Fig. 1:**
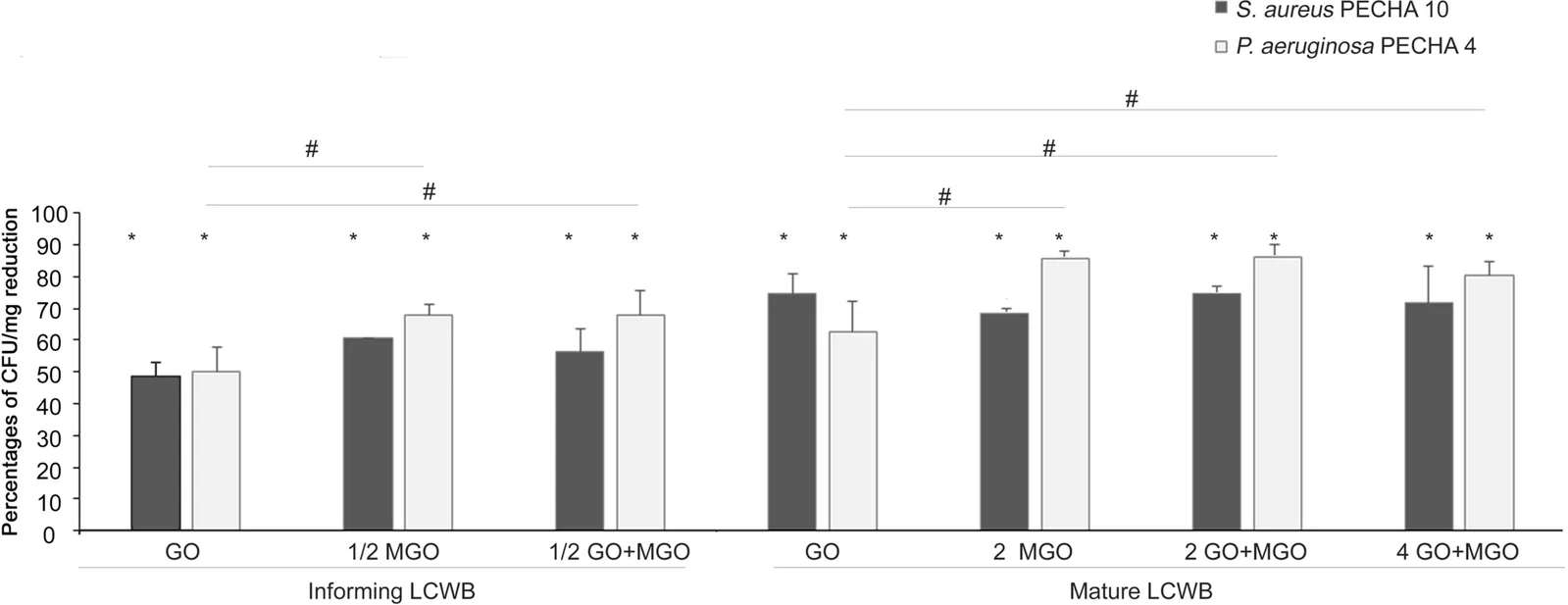
Percentage reduction of CFU/mg of *S. aureus* PECHA 10 and *P. aeruginosa* PECHA 4 in the LCWBs after treatment with: GO (50 mg/l), 1/2 MGO (16 mg/l), 1/2 GO + MGO (3.12 mg/l + 32 mg/l), 2 MGO (256 mg/l), 2 GO + MGO (12.5 mg/l + 128 mg/l) and 4 GO + MGO (25 mg/l + 256 mg/l). *Statistically significant (*p* < 0.05) in respect to the control; #statistically significant (*p* < 0.05) between each tested group

Figure 1 shows the percentage of CFU/mg biofilm reduction after treatment with GO (50 mg/l), MGO and additive combination of GO + MGO against both informing and mature LCWBs in respect to the untreated samples, whereas the caption in Fig. 1 specifies the concentrations used in the different combinations.

The GO and Amikacin data confirmed the previously published results (Di Giulio et al. 2020).

MGO at sub-MIC values showed significant CFU/mg reductions with respect to the control for both tested strains. In particular, in presence of 1/2 MIC of MGO, the percentages of biofilm reduction were 61.0% ± 7.8 and 56.2% ± 13.9 for *S. aureus* and *P. aeruginosa*, respectively. The GO + MGO combination, at sub-additive combination, showed a significant antibiofilm effect on informing LCWB. In presence of the additive combination, the CFU/mg reduction with respect to the control for *S. aureus* was 57.3% ± 8.8 and 64.0% ± 7.3 for *P. aeruginosa*. The GO + MGO combination increased the GO and MGO effect with relevant percentages of CFU/mg reduction in respect to the untreated samples (*p* < 0.05) and GO and MGO alone. Regarding the mature LCWB, 2 MGO showed a significant effect on *P. aeruginosa* (83.5% ± 7.9). For 2 GO + MGO, the percentage of CFU/mg reduction in respect to the control for each strain was 69.% ± 8.9 for *S. aureus* and 56.1% ± 28.8 for *P. aeruginosa*. In the case of mature LCWB, GO + MGO expressed a relevant effect on both bacteria despite the concentrations used being lower than the concentrations of the substances used individually.

**Fig. 2:**
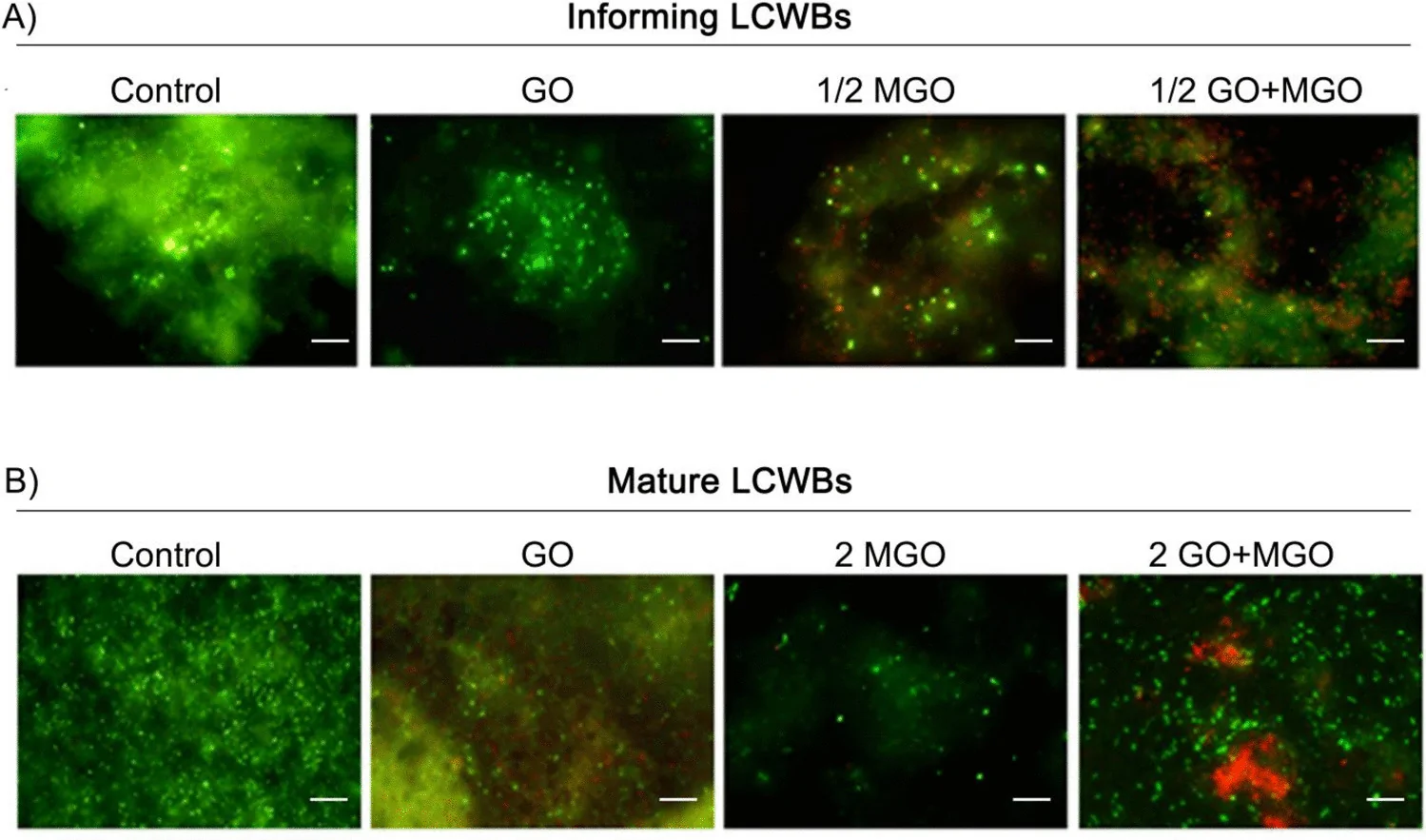
Representative Live/Dead images of informing (**A**) and mature (**B**) LCWBs after treatment with GO (50 mg/l), 1/2 MGO (16 mg/l), 1/2 GO + MGO (3.12 mg/l + 32 mg/l), 2 MGO (256 mg/l), 2 GO + MGO (12.5 mg/l + 128 mg/l) and 4 GO + MGO (25 mg/l + 256 mg/l). For informing LCWBs, GO, 1/2 MGO and 1/2 GO + MGO were added in the tubes and incubated for 48 h; for mature LCWBs, GO, 2 MGO and 2 GO + MGO were added at the 48 h-mature biofilm and incubated for 24 h. The green color refers to the viable bacterial cells while the red color refers to the dead cells. The images observed at fluorescent Leica 4000 DM microscopy are recorded at an excitation/emission wavelengths of 485/498 nm for SYTO 9 (green) and of 535/617 nm for Propidium iodide (red). Original magnification ×1000. Scale bar = 5 µm

Figure 2 shows representative Live/Dead images of informing and mature LCWBs after treatment with GO, MGO and GO + MGO. On informing LCWB, in presence of MGO (32 mg/l) a relevant number of dead (red) and disaggregated bacteria were detected in respect to the control. A combined antiadhesive and bacteriostatic effect of GO with a bactericidal effect of MGO was detected in presence of additive combination of GO + MGO (6.25 + 64 mg/l). Regarding the mature LCWB, in presence of MGO (128 mg/l) alone and combined with GO (6.25 mg/l + 64 mg/l), a higher number of viable (green) and disaggregated bacteria were displayed.

**Fig. 3:**
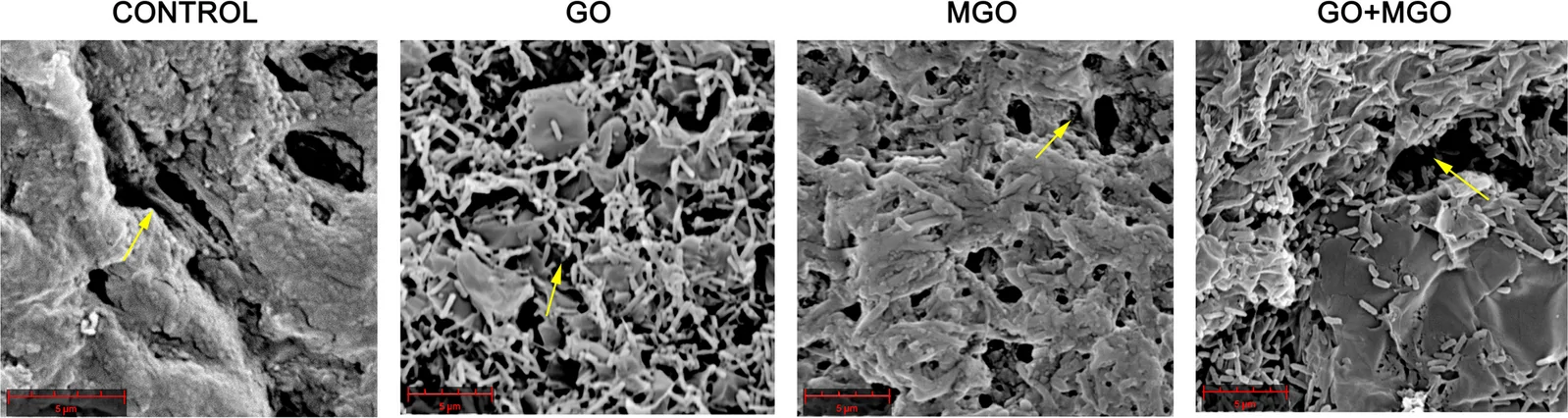
SEM images of mature LCWB treated with GO (50 mg/l), MGO (128 mg/l) and GO + MGO (6.25 mg/l + 64 mg/l). Yellow arrows indicate fibrin. Original magnification. 30 K. scale bar = 5µm

The SEM images (Fig. 3) demonstrate a greater effect on fibrin polymerization in the presence of GO + MGO combination. In presence of GO, a reduction in fibrin polymerization was more relevant than the control and MGO alone. When the LCWB was treated with GO + MGO combination, the fibrin and the microbial population were reduced. Fibrin appeared destructured in respect to the control and the sample treated with MGO.

**Fig. 4:**
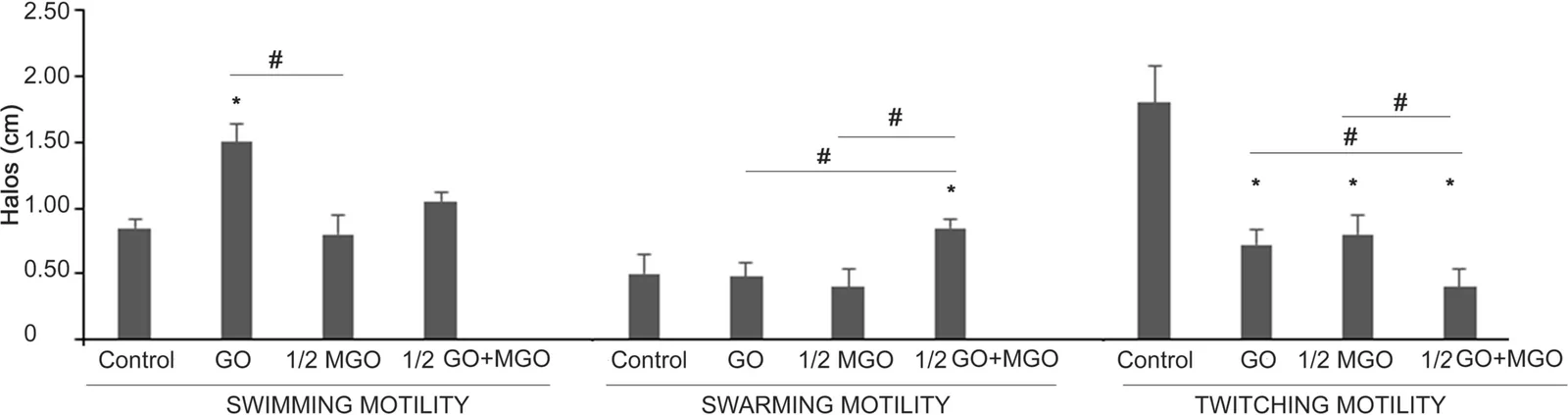
Halos diameters (cm) of *P. aeruginosa* PECHA 4 swimming, swarming and twitching motilities in presence of GO (50 mg/l), 1/2 MGO (64 mg/l), 1/2 GO + MGO (3.12 mg/l + 32 mg/l). *Statistically significant (*p* < 0.05) in respect to the control; # statistically significant (*p* < 0.05) between each tested group

As shown in Fig. 4, GO (50 mg/l) and 1/2 MGO (64 mg/l), either alone or in combination, did not significantly affect *P. aeruginosa* swimming and swarming motility, as the halo diameters were similar to or greater than those of the control. In contrast, GO and MGO, both individually and in combination, significantly reduced (*p* < 0.05) twitching motility compared with the control. Since twitching motility is closely associated with the initial stages of biofilm formation, the reduced halo diameter indicates a less capability of *P. aeruginosa* to form biofilms (Patel and Devarshi 2022). Although both compounds significantly reduced twitching motility when tested individually, the inhibitory effect was significantly greater when they were used in combination. Considering the role of twitching motility in the early stages of biofilm formation, this enhanced inhibition may contribute to the superior antibiofilm activity of the GO + MGO combination.

## Discussion

Chronic wound infections are difficult to treat due to polymicrobial biofilms that give high antimicrobial tolerance to antimicrobial agents. In fact, the biofilm plays an important role in wound healing as a physical barrier sheltering the embedded bacteria against the antimicrobial molecules and increasing the antimicrobial tolerance (Rawat and Gabrani 2025). In the biofilm, the microbial species establish a relationship of cooperation and competition evolving in a polymicrobial community that impairs wound healing more significantly than monomicrobial biofilm. The realized synergistic interaction among bacteria expresses different virulence phenotypes and more resistant behavior against traditional antimicrobial agents in respect to the planktonic phase (DeLeon et al. 2014; Serra et al. 2015).

In this study, we investigated the antimicrobial and antibiofilm activity of GO + MGO combination against polymicrobial biofilms composed of clinical isolates of *S. aureus* and *P. aeruginosa* in the LCWB model. The outcomes show that this non-antibiotic combination exerts enhanced antimicrobial activity, producing synergistic effects against *S. aureus,* additive effect against *P. aeruginosa,* remarkable antibiofilm effect and inhibition of *P.aeruginosa* twitching motility. Overall, the obtained results support the potential of this combination as an alternative strategy for chronic wound management.

GO antimicrobial effect has been extensively investigated and several studies have demonstrated its antibiofilm action (Lange et al. 2024; Saeed et al. 2023; Singh et al. 2025). In our previous study (Di Giulio et al. 2020), we demonstrated that GO was able to interfere with the coagulated structure produced by *S. aureus* with increased antimicrobial efficacy. In particular, it was observed the GO wrapping action on *S. aureus* cells due to electrostatic interactions, hydrogen bonding, and hydrophobic interactions that promote its membrane absorption (Ravikumar et al. 2022; Di Giulio et al. 2020). Furthermore, it has been recently demonstrated that GO is able to increase liposomal membrane fluidity due to its affinity for lipids (di Giacomo et al. 2026) and its ability to alter membrane integrity (Sengupta et al. 2019; Zhang et al. 2019; Chauhan et al. 2026). On the other hand, MGO displays an important antimicrobial action interfering with the *S. aureus* and *P. aeruginosa* growth (Hayes et al. 2018). The antimicrobial activity of MGO against *S. aureus* and *P. aeruginosa* has been attributed to the induction of cell lysis (Rabie et al. 2016). Moreover, MGO has been shown to reduce the efflux pump activity and increase the intracellular accumulation of linezolid in *S. aureus.* It has also exhibited significant synergism activity with piperacillin against *P. aeruginosa* (Hayes et al. 2018; Mukherjee et al. 2011). Furthermore, MGO generated by aerobic glycolysis in the activated macrophages showed to affect the bacterial infection by targeting bacterial proteins and DNA (Anaya-Sanchez et al. 2025). Therefore, the combination of GO with MGO may provide enhanced antimicrobial and antibiofilm effects through their complementary mechanisms of action.

Thus, GO and MGO exert antimicrobial activity through distinct and potentially complementary mechanisms, with GO mainly affecting the bacterial envelope, membrane properties, and biofilm architecture, and MGO acting through membrane damage and modification of bacterial proteins and DNA (Lange et al. 2024; Saeed et al. 2023; Singh et al. 2025; Diban et al 2025). Based on this rationale, a GO+MGO formulation for the treatment of infections associated with skin lesions was previously developed and patented (Italian Patent Application No. 102022000024408, “Composition for the treatment of infections affecting skin lesions — Composizione per il trattamento delle infezioni a carico delle lezioni cutanee”). The present study was therefore designed to provide an experimental characterization of the combined antimicrobial and antibiofilm activity of GO and MGO, with particular emphasis on their interaction against *S. aureus* and *P. aeruginosa* and on the mechanisms potentially underlying the effects of their combination.

Although, in the present study, cytotoxicity was not specifically assessed, previous studies have reported no significant cytotoxic effects of GO and MGO on different human cell models within comparable concentration ranges and under the experimental conditions investigated (Ettorre et al. 2016; D’Amico et al. 2025; Di Fermo et al. 2025). Notably, at the tested concentrations, in the present study, the GO+MGO combination exhibited marked antimicrobial and antibiofilm activity, with enhanced effects compared with those of the individual components (Di Giulio et al. 2020; Cebadero-Domínguez et al. 2022; Bengtson et al. 2016), potentially reflecting the complementary and multi-target mechanisms of action of GO and MGO.

A key finding of this study was the species-dependent interaction between GO and MGO, which resulted in synergistic antibacterial activity against *S. aureus* but only an additive effect against *P. aeruginosa*. This differential response may reflect the greater ability of *P. aeruginosa* to withstand the oxidative and carbonyl stress induced by MGO, owing to its robust antioxidant defenses, efficient glyoxalase systems, and remarkable metabolic plasticity (Vijayraghavan et al. 2025). These protective mechanisms may limit the ability of MGO activity by GO, resulting in an additive rather than synergistic interaction. Conversely, the synergistic effect observed against *S. aureus* may be linked to membrane perturbation. Membrane fluidity is critically dependent on phospholipid organization and fatty-acyl-chain conformation and can be markedly altered by antimicrobial compounds (Di Lodovico et al. 2020). Notably, MGO has been reported to increase membrane fluidity in *S. aureus*, and our findings similarly revealed a significant increase in membrane fluidity following treatment. Such membrane remodeling may increase bacterial susceptibility by compromising membrane-associated functions and facilitating GO interaction with the cell envelope and/or MGO access to intracellular targets. This mechanism could therefore contribute to the enhanced antibacterial activity of the GO+MGO combination against *S. aureus*. Nevertheless, the observed synergism is likely multifactorial, potentially arising from the simultaneous targeting of distinct cellular pathways, physicochemical interactions between GO and MGO, and interference with bacterial stress-response or resistance mechanisms (Brown 2015; Yoon et al. 2015).

Beyond their antibacterial activity against planktonic cells, GO and MGO exhibited pronounced antibiofilm activity against polymicrobial biofilms. In the LCWB model, MGO, either alone or in combination with GO, significantly reduced *S. aureus* and *P. aeruginosa* cell viable counts, as demonstrated by the decrease in CFU/mg of biofilm. The antibiofilm activity of the GO+MGO combination may arise from complementary mechanisms of action: GO may interfere with the structural organization of the biofilm and fibrin network formation, thereby disrupting biofilm architecture, while MGO may exert a more direct antimicrobial effect on *S. aureus* and *P. aeruginosa*. Consistent with this hypothesis, MGO treatment was associated with a particularly high proportion of dead cells, supporting a predominantly bactericidal activity under the experimental conditions used, in contrast to the mainly bacteriostatic effect observed with GO at the tested concentrations (Sengupta et al. 2019; Chauhan et al. 2026). The complementary activities of GO and MGO therefore suggest that their combination may represent a promising strategy for controlling polymicrobial communities associated with chronic wounds. In addition to its potential therapeutic application against established biofilms, the GO+MGO combination could also be explored as a preventive approach to limit bacterial establishment and the progression from wound contamination to persistent colonization (Cacaci et al. 2019; Lu et al. 2014).

In addition to its antibiofilm activity, the GO+MGO combination significantly impaired *P. aeruginosa* twitching motility, a type IV pili-dependent surface translocation mechanism that plays a key role in surface colonization and the early stages of biofilm development. Its inhibition may therefore limit bacterial spreading into deeper wound regions and interfere with the establishment of structured biofilm communities. Interestingly, GO+MGO treatment also favored the planktonic state of *P. aeruginosa*, while exerting no substantial effect on swimming or swarming motility. Collectively, these findings suggest that the GO+MGO combination may not only reduce established polymicrobial biofilms but also interfere with key processes involved in *P. aeruginosa* surface colonization and biofilm initiation, thereby shifting the bacterial population toward a more planktonic and potentially more treatment-susceptible phenotype.

## Conclusion

The GO and MGO combination represents a novel non-antibiotic strategy to control microbial proliferation in a chronic wound combining low concentrations, a favorable cytotoxicity profile, antimicrobial/antibiofilm actions (enhanced effect, superior to the sum of the individual components) and interesting complementary multitarget mechanisms of action.

The proposed combination is innovative because:

- To the best of our knowledge, this is the first study to investigate the combined antimicrobial and antibiofilm effects of GO and MGO in a chronic wound biofilm model;
- It uses GO not merely as a delivery system but as a biologically active compound with antimicrobial and antibiofilm properties;
- It integrates multi-target antimicrobial actions with non-antibiotic mechanisms that disrupt multiple cellular processes, thereby limiting the microbial capacity to develop resistance or tolerance, although this aspect requires further investigation. This aspect is crucial for selecting alternative compounds that retain intrinsic antimicrobial efficacy without resorting to antibiotic use;
- The eco-sustainability of the proposed compounds derives from production cycles that have no environmental impact and their easy availability.

Although the present findings are limited to in vitro models and require further validation in vivo, the GO + MGO combination demonstrated promising antimicrobial and antibiofilm activity against resistant bacterial strains isolated from chronic wounds and in the LCWB model, which reproduces key features of the polymicrobial organization and microenvironment of chronic wound biofilms. The complementary and multi-target activities of GO and MGO, together with their efficacy under wound-like experimental conditions, support the potential of this combination as a strategy for the local management of chronic wound-associated infections. These findings provide a rationale for its further development into advanced topical wound-care formulations, including functionalized dressings, creams, or ointments, while future in vivo studies will be required to establish its safety, efficacy, and translational potential. Overall, the present study provides experimental support for the GO+MGO combination previously proposed in Patent No. 102022000024408 and highlights its potential as a non-antibiotic approach to targeting polymicrobial biofilms associated with difficult to treat chronic wounds.

## Supplementary Information

Below is the link to the electronic supplementary material.

ESM 1 (DOCX 12.0 MB)

## Data Availability

No datasets were generated or analysed during the current study.

## References

[CR1] Almuhanna Y (2025) Microbial biofilms as barriers to chronic wound healing: diagnostic challenges and therapeutic advances. J Clin Med 14:8121. 10.3390/jcm14228121 PMC12653366 41303157

[CR2] Anaya-Sanchez A, Berry SB, Espich S, Zilinskas A, Tran PM, Agudelo C, Samani H, Darwin KH, Portnoy DA, Stanley SA (2025) Methylglyoxal is an antibacterial effector produced by macrophages during infection. Cell Host Microbe 33:1121–1132. 10.1016/j.chom.2025.05.026 PMC12245573 40555231

[CR3] Bengtson S, Kling K, Madsen AM, Noergaard AW, Jacobsen NR, Clausen PA, Alonso B, Pesquera A, Zurutuza A, Ramos R, Okuno H, Dijon J, Wallin H, Vogel U (2016) No cytotoxicity or genotoxicity of graphene and graphene oxide in murine lung epithelial FE1 cells *in vitro*. Environ Mol Mutagen 57:469–482. 10.1002/em.22017 PMC5084775 27189646

[CR4] Borges A, Calvo MLM, Vaz JA, Calhelha RC (2024) Enhancing wound healing: a comprehensive review of sericin and *Chelidonium majus* L. as potential dressings. Mater 17:4199. 10.3390/ma17174199 PMC11395905 39274589

[CR5] Brown D (2015) Antibiotic resistance breakers: can repurposed drugs fill the antibiotic discovery void? Nat Rev Drug Discov 14(12):821–832. 10.1038/nrd4675 26493767

[CR6] Bucekova M, Jardekova L, Juricova V, Bugarova V, Di Marco G, Gismondi A, Leonardi D, Farkasovska J, Godocikova J, Laho M, Klaudiny J, Majtan V, Canini A, Majtan J (2019) Antibacterial activity of different blossom honeys: new findings. Molecules 24:1573. 10.3390/molecules24081573 PMC6514785 31010070

[CR7] Cacaci M, Martini C, Guarino C, Torelli R, Bugli F, Sanguinetti M (2019) Graphene oxide coatings as tools to prevent microbial biofilm formation on medical device. Adv Exp Med Biol. 10.1007/5584_2019_434 31468360

[CR8] Cebadero-Domínguez O, Ferrández-Gómez B, Sánchez-Ballester S, Moreno J, Jos A, Cameán AM (2022) *In vitro* toxicity evaluation of graphene oxide and reduced graphene oxide on Caco-2 cells. Toxicol Rep 9:1130–1138. 10.1016/j.toxrep.2022.05.010 PMC9742864 36518447

[CR9] Chauhan NPS, Ashtari B, Eftekhari BS, Akhshik M, Maria HJ, Khosravimelal S, Seifalian N, Thomas S, Gholipourmalekabadi M, Seifalian AM (2026) Functionalization of graphene oxide and its applications in tissue engineering and regenerative medicine. Biomater Adv 178:214421. 10.1016/j.bioadv.2025 40752075

[CR10] CLSI Clinical and Laboratory Standards Institute (2019) Methods for antimicrobial susceptibility testing. 29th edn. M02, M07 and M011. CLSI, Wayne, PA, USA 31339681

[CR11] Combarros-Fuertes P, Estevinho LM, Teixeira-Santos R, Rodrigues AG, Pina-Vaz C, Fresno JM, Tornadijo ME (2019) Evaluation of physiological effects induced by manuka honey upon *Staphylococcus aureus* and *Escherichia coli*. Microorganisms 7:258. 10.3390/microorganisms7080258 PMC6722746 31412630

[CR12] D’Amico E, Pierfelice TV, D’Ercole L, Di Fermo P, Iezzi G, D’ercole S, Petrini M (2025) The synergistic effect of photobiomodulation, methylglyoxal, and complex magnetic fields on human dermal fibroblasts: potential applications for chronic wound treatments. Lasers Med Sci 40:517. 10.1007/s10103-025-04775-3 PMC12695920 41372538

[CR13] Deglovic J, Majtanova N, Majtan J (2022) Antibacterial and antibiofilm effect of honey in the prevention of dental caries: a recent perspective. Foods 11(17):2670. 10.3390/foods11172670 PMC9455747 36076855

[CR14] DeLeon S, Clinton A, Fowler H, Everett J, Horswill AR, Rumbaugh KP (2014) Synergistic interactions of *Pseudomonas aeruginosa* and *Staphylococcus aureus* in an* in vitro* wound model. Infect Immun 82(11):4718–4728. 10.1128/IAI.02198-14 PMC4249327 25156721

[CR15] Di Bella S, Luzzati R, Mearelli F, Papa G, Spazzapan L, Nunnari A, D’Aleo F, Papola C, Principe L (2024) Anti-infective management of infected skin ulcers. Infez Med 32(2):138–147. 10.53854/liim-3202-3 PMC11142418 38827836

[CR16] Di Giulio M, Di Lodovico S, Fontana A, Traini T, Di Campli E, Pilato S, D’Ercole S, Cellini L (2020) Graphene Oxide affects *Staphylococcus aureus* and *Pseudomonas aeruginosa* dual species biofilm in chronic wound biofilm Lubbock model. Sci Rep 10(1):18525. 10.1038/s41598-020-75086-6 PMC7595099 33116164

[CR17] Di Fermo P, Diban F, Di Campli E, Cellini L, Pinti M, Di Giulio M, Petrini M, D’Ercole S, Di Lodovico S (2025) Interkingdom biofilms are affected by non-antibiotic strategies: *in vitro* study in Lubbock Chronic Wound Biofilm model. Int J Mol Sci 26:11658. 10.3390/ijms262311658 PMC12692427 41373806

[CR18] di Giacomo S, Moffa S, Pilato S, Siani G, Ciulla M, Ramal-Sanchez M, Valbonetti L, Bernabò N, Barboni B, Said J, Ceja-Vega J, Lee S, Fontana A (2026) Study of the interaction between graphene oxide and cholesterol using different artificial membrane models. J Colloid Interface Sci 712:140105. 10.1016/j.jcis.2026 41707502

[CR19] Di Giulio M, Zappacosta R, Di Lodovico S, Di Campli E, Siani G, Fontana A, Cellini L (2018) Antimicrobial and antibiofilm efficacy of Graphene Oxide against chronic wound microorganisms. Antimicrob Agents Chemother 62:e00547-18. 10.1128/AAC.00547-18 PMC6021640 29661876

[CR20] Di Lodovico S, Menghini L, Ferrante C, Recchia E, Castro-Amorim J, Gameiro P, Cellini L, Bessa LJ (2020) Hop extract: an efficacious antimicrobial and anti-biofilm agent against multidrug-resistant Staphylococci strains and *Cutibacterium acnes*. Front Microbiol 11:1852. 10.3389/fmicb.2020.01852 PMC7438819 32903686

[CR21] Di Lodovico S, De Pasquale V, Nocera FP, Petrini M, Di Fermo P, Diban F, Pinti M, De Martino L, Tafuri S, Cellini L, Di Giulio M, D’Ercole S (2025) Recombinant NK1 protein and LEDs: an innovative strategy to counteract resistant *Staphylococcus pseudintermedius* and *Pseudomonas aeruginosa* strains. Probiotics Antimicrob Proteins 18:2599–2611. 10.1007/s12602-025-10702-3 PMC13013161 40788453

[CR22] Diban F, Di Lodovico S, Di Fermo P, D’Ercole S, D’Arcangelo S, Di Giulio M, Cellini L (2023) Biofilms in chronic wound infections: innovative antimicrobial approaches using the *in vitro* Lubbock Chronic Wound Biofilm Model. Int J Mol Sci 24(2):1004. 10.3390/ijms24021004 PMC9862456 36674518

[CR23] Diban F, Di Fermo P, Di Lodovico S, Petrini M, Pilato S, Fontana A, Pinti M, Di Giulio M, Lence E, González-Bello C, Cellini L, D’Ercole S (2025) Methylglyoxal alone or combined with light-emitting diodes/complex electromagnetic fields represent an effective response to microbial chronic wound infections. Antibiotics (Basel) 14(4):396. 10.3390/antibiotics14040396 PMC12024167 40298537

[CR24] Edwin ER, Sakthi Sanjana D, Michael B, Kathiresan S, Elumalai K, Jayaprakash N (2025) Metallic nanoparticle-functionalized graphene oxide nanocomposites for wound healing: a comprehensive review. Biomed Mater Devices. 10.1007/s44174-025-00411-4

[CR25] Eriksson E, Liu PY, Schultz GS, Martins‐Green MM, Tanaka R, Weir D, Gould LJ, Armstrong DG, Gibbons GW, Wolcott R, Olutoye OO, Kirsner RS, Gurtner GC (2022) Chronic wounds: treatment consensus. Wound Repair Regen 30(2):156–171. 10.1111/wrr.12994 PMC9305950 35130362

[CR26] Ersanli C, Tzora A, Voidarou CC, Skoufos S, Zeugolis DI, Skoufos I (2023) Biodiversity of skin microbiota as an important biomarker for wound healing. Biology (Basel) 12(9):1187. 10.3390/biology12091187 PMC10525143 37759587

[CR27] Ettorre V, De Marco P, Zara S, Perrotti V, Scarano A, Di Crescenzo A, Petrini M, Hadad C, Bosco D, Zavan B, Valbonetti L, Spoto G, Iezzi G, Piattelli A, Cataldi A, Fontana A (2016) *In vitro* and *in vivo* characterization of graphene oxide coated porcine bone granules. Carbon N Y 103:291–298. 10.1016/j.carbon.2016.03.010

[CR28] Graves N, Phillips CJ, Harding K (2021) A narrative review of the epidemiology and economics of chronic wounds. Br J Dermatol 187(2):141–148. 10.1111/bjd.20692 34549421

[CR29] Gurunathan S, Han JW, Dayem AA, Eppakayala V, Kim JH (2012) Oxidative stress-mediated antibacterial activity of graphene oxide and reduced graphene oxide in *Pseudomonas aeruginosa*. Int J Nanomedicine 7:5901–5914. 10.2147/IJN.S37397 PMC3514835 23226696

[CR30] Hayes G, Wright N, Gardner SL, Telzrow CL, Wommack AJ, Vigueira PA (2018) Manuka honey and methylglyoxal increase the sensitivity of *Staphylococcus aureus* to linezolid. Lett Appl Microbiol 66(6):491–495. 10.1111/lam.12880 29575121

[CR31] Ibberson CB, Barraza JP, Holmes AL, Cao P, Whiteley M (2022) Precise spatial structure impacts antimicrobial susceptibility of *S. aureus* in poly-microbial wound infections. Proc Natl Acad Sci U S A 119(51):e2212340119. 10.1073/pnas.2212340119 PMC9907066 36520668

[CR32] Kucera J, Sojka M, Pavlik V, Szuszkiewicz K, Velebny V, Klein P (2014) Multispecies biofilm in an artificial wound bed-A novel model for *in vitro* assessment of solid antimicrobial dressings. J Microbiol Methods 103:18–24. 10.1016/j.mimet.2014.05.008 24880129

[CR33] Lange A, Kutwin M, Zawadzka K, Ostrowska A, Strojny-Cieślak B, Nasiłowska B, Bombalska A, Jaworski S (2024) Impaired biofilm development on graphene oxide-metal nanoparticle composites. Nanotechnol Sci Appl 17:303–320. 10.2147/NSA.S485841 PMC11681909 39734361

[CR34] Lee C, Park C (2017) Bacterial responses to glyoxal and methylglyoxal: reactive electrophilic species. Int J Mol Sci 18(1):169. 10.3390/ijms18010169 PMC5297802 28106725

[CR35] Liu S, Zeng TH, Hofmann M, Burcombe E, Wei J, Jiang R, Kong J, Chen Y (2011) Antibacterial activity of graphite, graphite oxide, graphene oxide, and reduced graphene oxide: membrane and oxidative stress. ACS Nano 5(9):6971–6980. 10.1021/nn202451x 21851105

[CR36] Lu J, Turnbull L, Burke CM, Liu M, Carter DA, Schlothauer RC, Whitchurch CB, Harry EJ (2014) Manuka-type honeys can eradicate biofilms produced by *Staphylococcus aureus* strains with different biofilm-forming abilities. PeerJ 2:e326. 10.7717/peerj.326 PMC3970805 24711974

[CR37] Majtan J (2020) Honey: an ancient wound remedy and modern drug for wound care. Wound Repair Regen 28:419–424. 10.1111/wrr.12869

[CR38] McArthur A, Elsom RL, Marczylo T, O’Connor R, Dockrell M, Sturgeon K (1997) Committee on Toxicity of Chemical in Food, consumer products and the environment (Art A) Committee on Toxicity of Chemical in Food, consumer products and the environment on FSA funded project on evaluation and expression of uncertainty in risk assessment

[CR39] Mukherjee S, Chaki S, Das S, Sen S, Dutta SK, Dastidar SG (2011) Distinct synergistic action of piperacillin and methylglyoxal against *Pseudomonas aeruginosa*. Indian J Exp Biol 49:547–551 21800506

[CR40] Norman G, Christie J, Liu Z, Westby MJ, Jefferies JM, Hudson T, Edwards J, Mohapatra DP, Hassan IA, Dumville JC (2017) Antiseptics for burns. Cochrane Database Syst Rev 7(7):CD011821. 10.1002/14651858.CD011821.pub2 PMC6483239 28700086

[CR41] Odds FC (2003) Synergy, antagonism, and what the checkerboard puts between them. J Antimicrob Chemother 52:1. 10.1093/jac/dkg301 12805255

[CR42] Paramasivan S, Drilling AJ, Jardeleza C, Jervis-Bardy J, Vreugde S, Wormald PJ (2014) Methylglyoxal-augmented manuka honey as a topical anti–*Staphylococcus aureus* biofilm agent: safety and efficacy in an *in vivo *model. Int Forum Allergy Rhinol 4:187–195. 10.1002/alr.21264 24415444

[CR43] Park S, Lee KS, Bozoklu G, Cai W, Nguyen ST, Ruoff RS (2008) Graphene oxide papers modified by divalent ions—enhancing mechanical properties via chemical cross-linking. ACS Nano 2(3):572–578. 10.1021/nn700349a 19206584

[CR44] Patel H, Devarshi G (2022) Cell adhesion and twitching motility influence strong biofilm formation in *Pseudomonas aeruginosa*. Biofouling 38:235–249. 10.1080/08927014.2022.2054703 35345952

[CR45] Pouget C, Dunyach-Remy C, Magnan C, Pantel A, Sotto A, Lavigne JP (2022) Poly-microbial biofilm organization of *Staphylococcus aureus* and *Pseudomonas aeruginosa* in a chronic wound environment. Int J Mol Sci 23:10761. 10.3390/ijms231810761 PMC9504628 36142675

[CR46] Rabie E, Serem JC, Oberholzer HM, Gaspar AR, Bester MJ (2016) How methylglyoxal kills bacteria: an ultrastructural study. Ultrastruct Pathol 40:107–11. 10.3109/01913123.2016.1154914 26986806

[CR47] Ravikumar V, Mijakovic I, Pandit S (2022) Antimicrobial activity of graphene oxide contributes to alteration of key stress-related and membrane bound proteins. Int J Nanomedicine 17:6707–6721. 10.2147/IJN.S387590 PMC9805717 36597432

[CR48] Rawat K, Gabrani R (2025) Phytotherapy for chronic wound management in the era of antibiotic resistance. Wounds 37:363–372. 10.25270/wnds/24210 41176770

[CR49] Roberts AEL, Maddocks SE, Cooper RA (2021) Manuka honey is bactericidal against antibiotic-resistant bacteria via multiple mechanisms. Front Microbiol 12:660466. 10.3389/fmicb.2021.660466

[CR50] Saeed SI, Vivian L, Zalati CWSCW, Sani NIM, Aklilu E, Mohamad M, Noor AAM, Muthoosamy K, Kamaruzzaman NF (2023) Antimicrobial activities of graphene oxide against biofilm and intracellular *Staphylococcus aureus* isolated from bovine mastitis. BMC Vet Res 19:10. 10.1186/s12917-022-03560 PMC9840331 36641476

[CR51] Sen CK (2021) Human wound and its burden: updated 2020 compendium of estimates. Adv Wound Care (New Rochelle) 10(5):281–292. 10.1089/wound.2021.0026 PMC8024242 33733885

[CR52] Sengupta I, Bhattacharya P, Talukdar M, Neogi S, Pal SK, Chakraborty S (2019) Bactericidal effect of graphene oxide and reduced graphene oxide: influence of shape of bacteria. Colloid Interface Sci Commun 28:60–68. 10.1016/j.colcom.2018.12.001

[CR53] Serra R, Grande R, Butrico L, Rossi A, Settimio UF, Caroleo B, Amato B, Gallelli L, de Franciscis S (2015) Chronic wound infections: the role of *Pseudomonas aeruginosa* and *Staphylococcus aureus*. Expert Rev Anti Infect Ther 13(5):605–613. 10.1586/14787210.2015.1023291 25746414

[CR54] Singh A, Mahapatra SN, Mitra S, Yadav N, Lochab B, Mukhopadhyay K (2025) Antibacterial and antibiofilm efficacy of graphene oxide nanomaterials against gram-positive bacteria: mechanistic approaches to understand the membrane damage and oxidative stress in cells. Langmuir 41:29516–29527. 10.1021/acs.langmuir.5c03508 41150896

[CR55] Thaarup IC, Bjarnsholt T (2021) Current *in vitro* biofilm-infected chronic wound models for developing new treatment possibilities. Adv Wound Care (New Rochelle) 10:91–102. 10.1089/wound.2020.1176 32496982

[CR56] Tottoli EM, Dorati R, Genta I, Chiesa E, Pisani S, Conti B (2020) Skin wound healing process and new emerging technologies for skin wound care and regeneration. Pharmaceutics 12:735. 10.3390/pharmaceutics12080735 PMC7463929 32764269

[CR57] Uberoi A, McCready-Vangi A, Grice EA (2024) The wound microbiota: microbial mechanisms of impaired wound healing and infection. Nat Rev Microbiol 22(8):507–521. 10.1038/s41579-024-01035-z 38575708

[CR58] Vestweber PK, Wächter J, Planz V, Jung N, Windbergs M (2024) The interplay of *Pseudomonas aeruginosa* and *Staphylococcus aureus* in dual-species biofilms impacts development, antibiotic resistance and virulence of biofilms in *in vitro* wound infection models. PLoS ONE 19(5):e0304491. 10.1371/journal.pone.0304491 PMC11132468 38805522

[CR59] Vijayraghavan S, Ruggiero A, Becker S, Mieczkowski P, Hanna GS, Hamann MT, Saini N (2025) Methylglyoxal mutagenizes single-stranded DNA via Rev1-associated slippage and mispairing. Nucleic Acids Res 53:gkaf705. 10.1093/nar/gkaf705 PMC12288878 40705923

[CR60] Yoon Y, Lee H, Lee S, Kim S, Choi KH (2015) Membrane fluidity-related adaptive response mechanisms of foodborne bacterial pathogens under environmental stresses. Food Res Int 72:25–36. 10.1016/j.foodres.2015.03.016

[CR61] Zhang X, Cao F, Wu L, Jiang X (2019) Understanding the synergic mechanism of weak interactions between graphene oxide and lipid membrane leading to the extraction of lipids. Langmuir 35(43):14098–14107. 10.1021/acs.langmuir.9b02536 31594302

