# Supplementary material for: Graphene Oxide and Methylglyoxal: a combined strategy against chronic wound pathogens
Source: Appl Microbiol Biotechnol. 2026 Sep 24;110:288. doi: 10.1007/s00253-026-14050-2 (PMC13615202; doi:10.1007/s00253-026-14050-2)
Supplement: Supplementary file 1 [file 253_2026_14050_MOESM1_ESM.docx]

**Graphene Oxide and Methylglyoxal: A Combined Strategy Against Chronic Wound Pathogens**

Silvia Di Lodovico^1*^, Antonella Fontana^1^, Paola Di Fermo^2^, Firas Diban^1^, Emanuela Di Campli^1^, Serena Pilato^1^, Simonetta D’Ercole^2^, Luigina Cellini^1^, Mara Di Giulio^1^

^1^Department of Pharmacy, University “G. d’Annunzio” Chieti-Pescara, Via dei Vestini, 31, 66100 Chieti, Italy ^2^Department of Medical, Oral and Biotechnological Sciences, University "G. d'Annunzio" Chieti-Pescara, Via dei Vestini, 31, 66100 Chieti, Italy.

*Corresponding author: Silvia Di Lodovico

Mail: silvia.dilodovico@unich.it

**SUPPORTING INFORMATION**

**Figure S1.** Raman (A) and FTIR (B) spectroscopy analyses of GO dispersion before and after UVC sterilization treatment. Transmittance and Raman intensity units are not reported because the spectra were normalized and overlaid to facilitate direct comparison.

Raman spectra of both untreated GO (black line) and UVC-treated GO (red line) exhibited the characteristic D and G peaks centered at ~1350 and ~1590 cm^-1^, respectively (Figure S1A). The D band is associated with structural defects and disorder within the carbon lattice, whereas the G band arises from the in-plane vibration of *sp^2^*-hybridized carbon atoms. The overall spectral profiles of the two samples were nearly superimposable with no appreciable shifts in band position of the D and G bands after UVC exposure. The FTIR spectra further confirmed the preservation of GO chemical functionalities following UVC treatment (Figure S1B). Both samples displayed the characteristic broad O-H stretching band around 3200 cm^-1^, the C=O stretching vibration at approximately 1724 cm^-1^, the C=C skeletal vibration near 1620 cm^-1^, and the C-O-C stretching band around 1075 cm^-1^. The presence and relative intensity of these absorption bands were maintained after UVC sterilization, indicating that the oxygen-containing functional groups characteristic of GO remained substantially unaltered. Minor variations in band intensity were observed between the spectra; however, these differences were within the experimental variability of the measurements and did not reveal any statistically significant change attributable to the UVC treatment.
